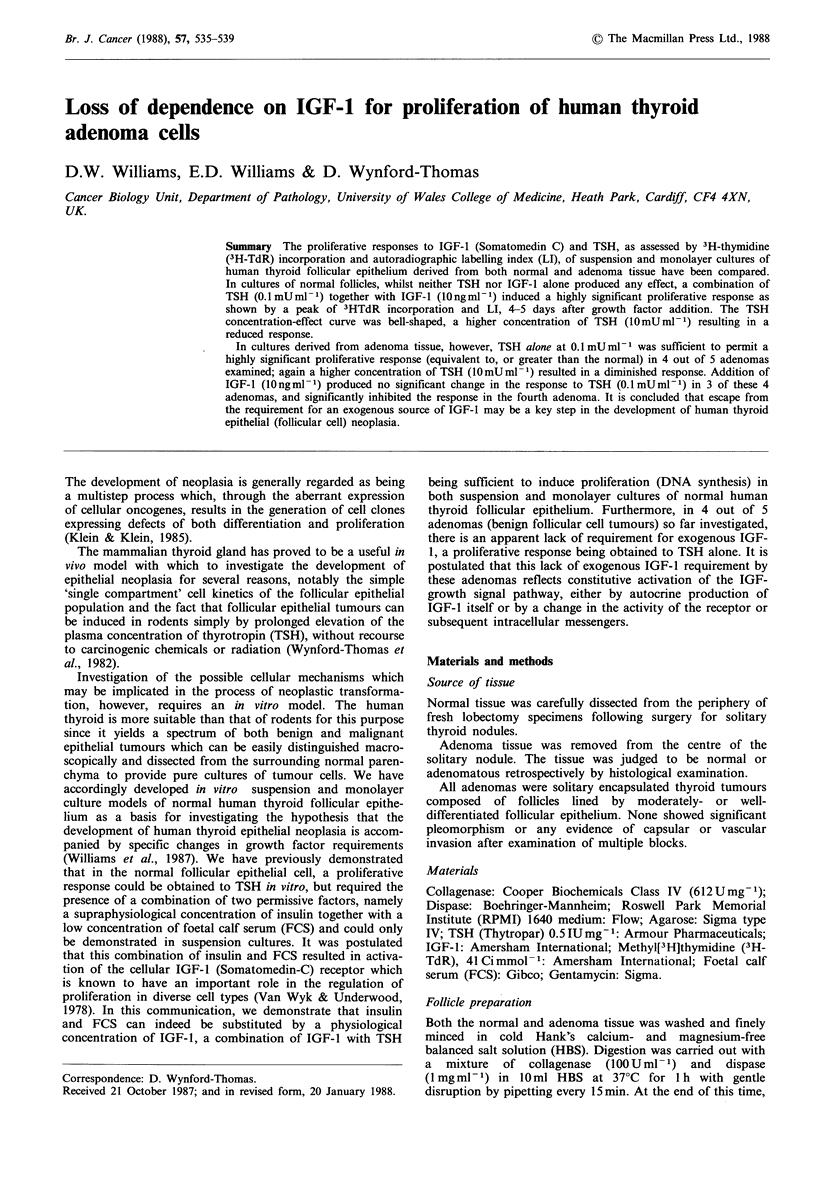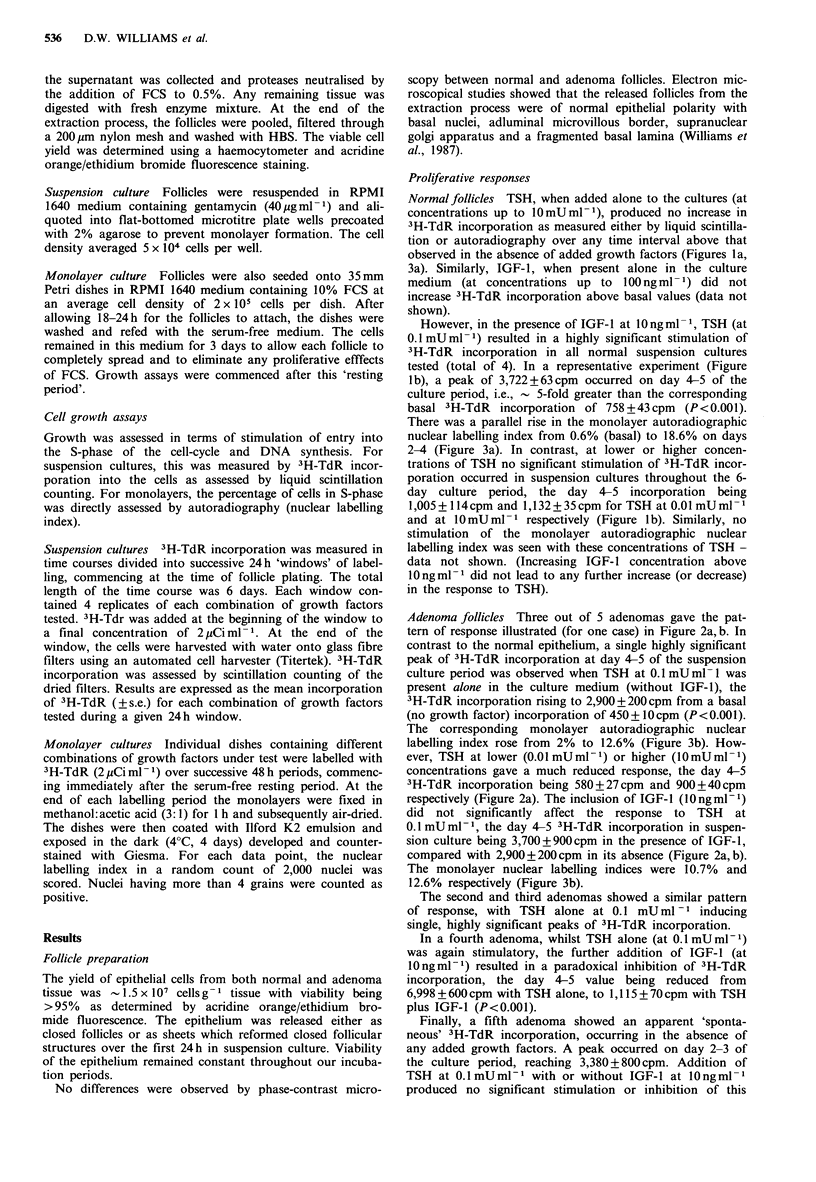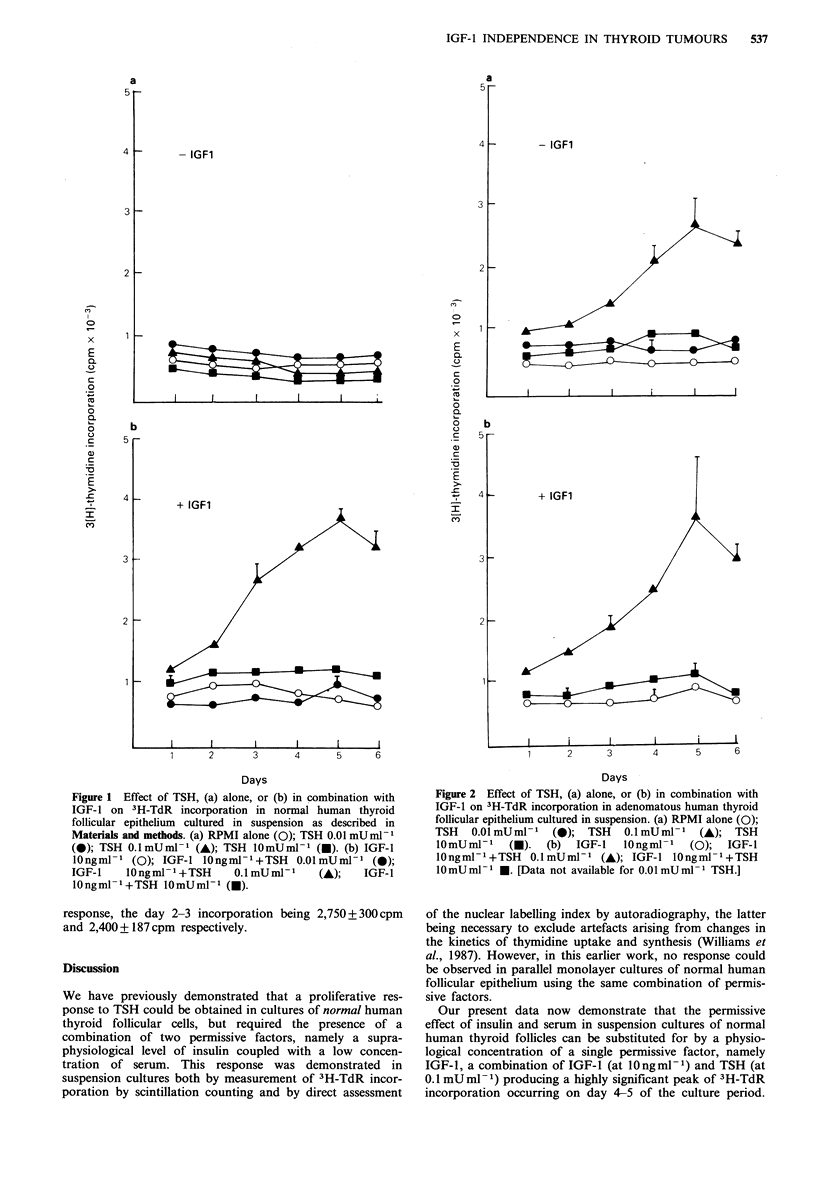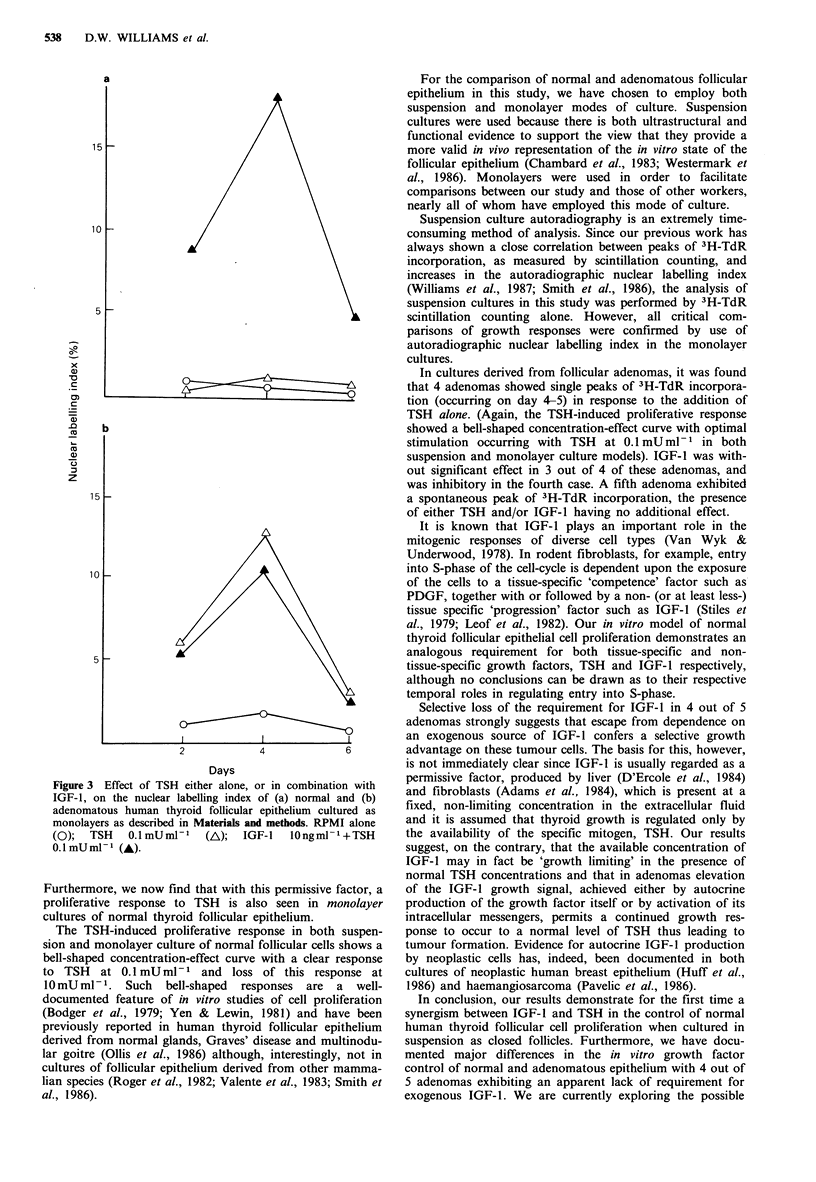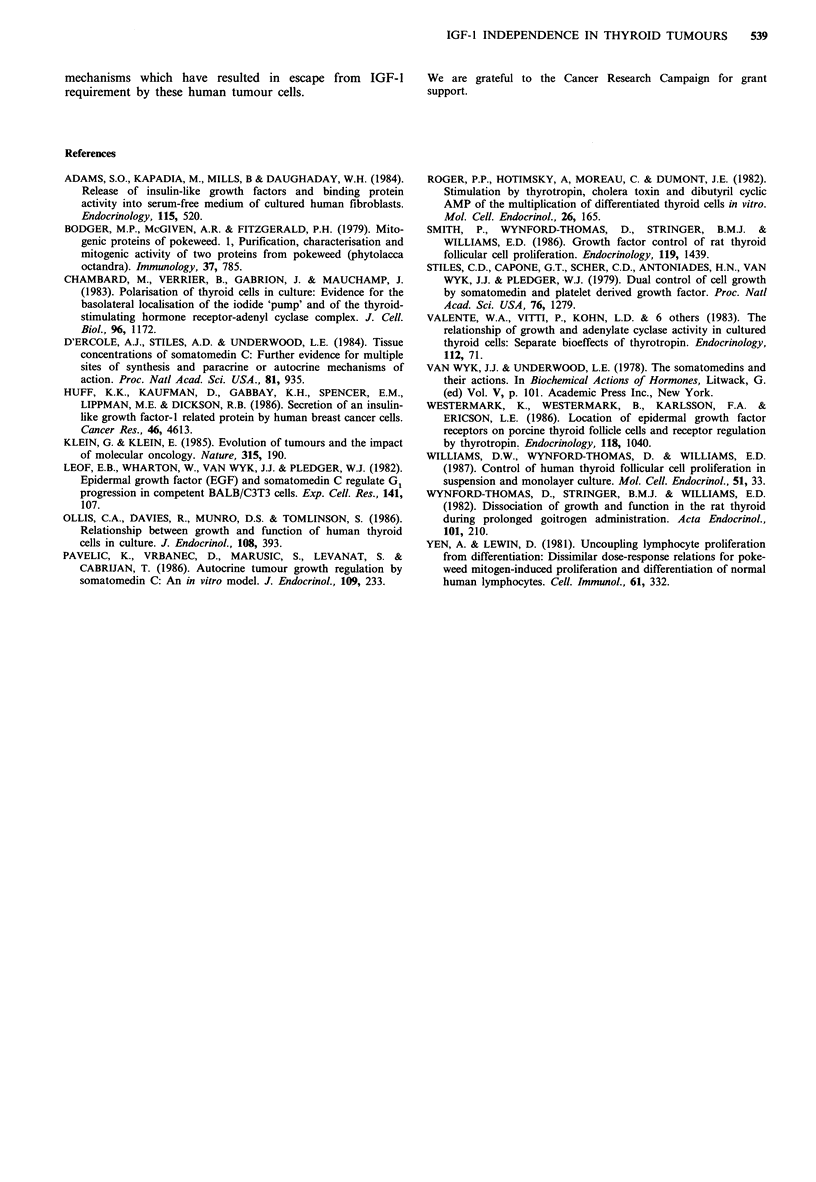# Loss of dependence on IGF-1 for proliferation of human thyroid adenoma cells.

**DOI:** 10.1038/bjc.1988.124

**Published:** 1988-06

**Authors:** D. W. Williams, E. D. Williams, D. Wynford-Thomas

**Affiliations:** Department of Pathology, University of Wales College of Medicine, Cardiff, UK.

## Abstract

The proliferative responses to IGF-1 (Somatomedin C) and TSH, as assessed by 3H-thymidine (3H-TdR) incorporation and autoradiographic labelling index (LI), of suspension and monolayer cultures of human thyroid follicular epithelium derived from both normal and adenoma tissue have been compared. In cultures of normal follicles, whilst neither TSH nor IGF-1 alone produced any effect, a combination of TSH (0.1 mU ml-1) together with IGF-1 (10 ng ml-1) induced a highly significant proliferative response as shown by a peak of 3HTdR incorporation and LI, 4-5 days after growth factor addition. The TSH concentration-effect curve was bell-shaped, a higher concentration of TSH (10 mU ml-1) resulting in a reduced response. In cultures derived from adenoma tissue, however, TSH alone at 0.1 mU ml-1 was sufficient to permit a highly significant proliferative response (equivalent to, or greater than the normal) in 4 out of 5 adenomas examined; again a higher concentration of TSH (10 mU ml-1) resulted in a diminished response. Addition of IGF-1 (10 ng ml-1) produced no significant change in the response to TSH (0.1 mU ml-1) in 3 of these 4 adenomas, and significantly inhibited the response in the fourth adenoma. It is concluded that escape from the requirement for an exogenous source of IGF-1 may be a key step in the development of human thyroid epithelial (follicular cell) neoplasia.


Br. J. Cancer (1988), 57, 535–539

© The Macmillan Press Ltd., 1988

### Loss of dependence on IGF-1 for proliferation of human thyroid adenoma cells

D.W. Williams, E.D. Williams & D. Wynford-Thomas

*Cancer Biology Unit, Department of Pathology, University of Wales College of Medicine, Heath Park, Cardiff, CF4 4XN, UK.*

**Summary** The proliferative responses to IGF-1 (Somatomedin C) and TSH, as assessed by $^{3}$H-thymidine ($^{3}$H-TdR) incorporation and autoradiographic labelling index (LI), of suspension and monolayer cultures of human thyroid follicular epithelium derived from both normal and adenoma tissue have been compared. In cultures of normal follicles, whilst neither TSH nor IGF-1 alone produced any effect, a combination of TSH (0.1 mU ml$^{-1}$) together with IGF-1 (10 ng ml$^{-1}$) induced a highly significant proliferative response as shown by a peak of $^{3}$HTdR incorporation and LI, 4–5 days after growth factor addition. The TSH concentration-effect curve was bell-shaped, a higher concentration of TSH (10 mU ml$^{-1}$) resulting in a reduced response.

In cultures derived from adenoma tissue, however, TSH *alone* at 0.1 mU ml$^{-1}$ was sufficient to permit a highly significant proliferative response (equivalent to, or greater than the normal) in 4 out of 5 adenomas examined; again a higher concentration of TSH (10 mU ml$^{-1}$) resulted in a diminished response. Addition of IGF-1 (10 ng ml$^{-1}$) produced no significant change in the response to TSH (0.1 mU ml$^{-1}$) in 3 of these 4 adenomas, and significantly inhibited the response in the fourth adenoma. It is concluded that escape from the requirement for an exogenous source of IGF-1 may be a key step in the development of human thyroid epithelial (follicular cell) neoplasia.

The development of neoplasia is generally regarded as being a multistep process which, through the aberrant expression of cellular oncogenes, results in the generation of cell clones expressing defects of both differentiation and proliferation (Klein & Klein, 1985).

The mammalian thyroid gland has proved to be a useful *in vivo* model with which to investigate the development of epithelial neoplasia for several reasons, notably the simple 'single compartment' cell kinetics of the follicular epithelial population and the fact that follicular epithelial tumours can be induced in rodents simply by prolonged elevation of the plasma concentration of thyrotropin (TSH), without recourse to carcinogenic chemicals or radiation (Wynford-Thomas *et al.*, 1982).

Investigation of the possible cellular mechanisms which may be implicated in the process of neoplastic transformation, however, requires an *in vitro* model. The human thyroid is more suitable than that of rodents for this purpose since it yields a spectrum of both benign and malignant epithelial tumours which can be easily distinguished macroscopically and dissected from the surrounding normal parenchyma to provide pure cultures of tumour cells. We have accordingly developed *in vitro* suspension and monolayer culture models of normal human thyroid follicular epithelium as a basis for investigating the hypothesis that the development of human thyroid epithelial neoplasia is accompanied by specific changes in growth factor requirements (Williams *et al.*, 1987). We have previously demonstrated that in the normal follicular epithelial cell, a proliferative response could be obtained to TSH *in vitro*, but required the presence of a combination of two permissive factors, namely a supraphysiological concentration of insulin together with a low concentration of foetal calf serum (FCS) and could only be demonstrated in suspension cultures. It was postulated that this combination of insulin and FCS resulted in activation of the cellular IGF-1 (Somatomedin-C) receptor which is known to have an important role in the regulation of proliferation in diverse cell types (Van Wyk & Underwood, 1978). In this communication, we demonstrate that insulin and FCS can indeed be substituted by a physiological concentration of IGF-1, a combination of IGF-1 with TSH being sufficient to induce proliferation (DNA synthesis) in both suspension and monolayer cultures of normal human thyroid follicular epithelium. Furthermore, in 4 out of 5 adenomas (benign follicular cell tumours) so far investigated, there is an apparent lack of requirement for exogenous IGF-1, a proliferative response being obtained to TSH alone. It is postulated that this lack of exogenous IGF-1 requirement by these adenomas reflects constitutive activation of the IGF-growth signal pathway, either by autocrine production of IGF-1 itself or by a change in the activity of the receptor or subsequent intracellular messengers.

Correspondence: D. Wynford-Thomas.
Received 21 October 1987; and in revised form, 20 January 1988.

### Materials and methods

### *Source of tissue*

Normal tissue was carefully dissected from the periphery of fresh lobectomy specimens following surgery for solitary thyroid nodules.

Adenoma tissue was removed from the centre of the solitary nodule. The tissue was judged to be normal or adenomatous retrospectively by histological examination.

All adenomas were solitary encapsulated thyroid tumours composed of follicles lined by moderately- or well-differentiated follicular epithelium. None showed significant pleomorphism or any evidence of capsular or vascular invasion after examination of multiple blocks.

### *Materials*

Collagenase: Cooper Biochemicals Class IV (612 U mg$^{-1}$); Dispase: Boehringer-Mannheim; Roswell Park Memorial Institute (RPMI) 1640 medium: Flow; Agarose: Sigma type IV; TSH (Thytropar) 0.5 IU mg$^{-1}$: Armour Pharmaceuticals; IGF-1: Amersham International; Methyl[$^{3}$H]thymidine ($^{3}$H-TdR), 41 Ci mmol$^{-1}$: Amersham International; Foetal calf serum (FCS): Gibco; Gentamycin: Sigma.

### *Follicle preparation*

Both the normal and adenoma tissue was washed and finely minced in cold Hank's calcium- and magnesium-free balanced salt solution (HBS). Digestion was carried out with a mixture of collagenase (100 U ml$^{-1}$) and dispase (1 mg ml$^{-1}$) in 10 ml HBS at 37°C for 1 h with gentle disruption by pipetting every 15 min. At the end of this time, the supernatant was collected and proteases neutralised by the addition of FCS to 0.5%. Any remaining tissue was digested with fresh enzyme mixture. At the end of the extraction process, the follicles were pooled, filtered through a 200 $\mu$m nylon mesh and washed with HBS. The viable cell yield was determined using a haemocytometer and acridine orange/ethidium bromide fluorescence staining.

*Suspension culture* Follicles were resuspended in RPMI 1640 medium containing gentamycin (40 $\mu g\,ml^{-1}$) and aliquoted into flat-bottomed microtitre plate wells precoated with 2% agarose to prevent monolayer formation. The cell density averaged $5 \times 10^4$ cells per well.

*Monolayer culture* Follicles were also seeded onto 35 mm Petri dishes in RPMI 1640 medium containing 10% FCS at an average cell density of $2 \times 10^5$ cells per dish. After allowing 18–24 h for the follicles to attach, the dishes were washed and refed with the serum-free medium. The cells remained in this medium for 3 days to allow each follicle to completely spread and to eliminate any proliferative efffects of FCS. Growth assays were commenced after this 'resting period'.

*Cell growth assays*

Growth was assessed in terms of stimulation of entry into the S-phase of the cell-cycle and DNA synthesis. For suspension cultures, this was measured by $^3$H-TdR incorporation into the cells as assessed by liquid scintillation counting. For monolayers, the percentage of cells in S-phase was directly assessed by autoradiography (nuclear labelling index).

*Suspension cultures* $^3$H-TdR incorporation was measured in time courses divided into successive 24 h 'windows' of labelling, commencing at the time of follicle plating. The total length of the time course was 6 days. Each window contained 4 replicates of each combination of growth factors tested. $^3$H-Tdr was added at the beginning of the window to a final concentration of 2 $\mu Ci\,ml^{-1}$. At the end of the window, the cells were harvested with water onto glass fibre filters using an automated cell harvester (Titertek). $^3$H-TdR incorporation was assessed by scintillation counting of the dried filters. Results are expressed as the mean incorporation of $^3$H-TdR ($\pm$s.e.) for each combination of growth factors tested during a given 24 h window.

*Monolayer cultures* Individual dishes containing different combinations of growth factors under test were labelled with $^3$H-TdR (2 $\mu Ci\,ml^{-1}$) over successive 48 h periods, commencing immediately after the serum-free resting period. At the end of each labelling period the monolayers were fixed in methanol:acetic acid (3:1) for 1 h and subsequently air-dried. The dishes were then coated with Ilford K2 emulsion and exposed in the dark (4°C, 4 days) developed and counterstained with Giesma. For each data point, the nuclear labelling index in a random count of 2,000 nuclei was scored. Nuclei having more than 4 grains were counted as positive.

### Results

*Follicle preparation*

The yield of epithelial cells from both normal and adenoma tissue was $\sim 1.5 \times 10^7$ cells g$^{-1}$ tissue with viability being >95% as determined by acridine orange/ethidium bromide fluorescence. The epithelium was released either as closed follicles or as sheets which reformed closed follicular structures over the first 24 h in suspension culture. Viability of the epithelium remained constant throughout our incubation periods.

No differences were observed by phase-contrast microscopy between normal and adenoma follicles. Electron microscopical studies showed that the released follicles from the extraction process were of normal epithelial polarity with basal nuclei, adluminal microvillous border, supranuclear golgi apparatus and a fragmented basal lamina (Williams *et al.*, 1987).

*Proliferative responses*

*Normal follicles* TSH, when added alone to the cultures (at concentrations up to 10 mU ml$^{-1}$), produced no increase in $^3$H-TdR incorporation as measured either by liquid scintillation or autoradiography over any time interval above that observed in the absence of added growth factors (Figures 1a, 3a). Similarly, IGF-1, when present alone in the culture medium (at concentrations up to 100 ng ml$^{-1}$) did not increase $^3$H-TdR incorporation above basal values (data not shown).

However, in the presence of IGF-1 at 10 ng ml$^{-1}$, TSH (at 0.1 mU ml$^{-1}$) resulted in a highly significant stimulation of $^3$H-TdR incorporation in all normal suspension cultures tested (total of 4). In a representative experiment (Figure 1b), a peak of 3,722 $\pm$ 63 cpm occurred on day 4–5 of the culture period, i.e., $\sim$ 5-fold greater than the corresponding basal $^3$H-TdR incorporation of 758 $\pm$ 43 cpm ($P<0.001$). There was a parallel rise in the monolayer autoradiographic nuclear labelling index from 0.6% (basal) to 18.6% on days 2–4 (Figure 3a). In contrast, at lower or higher concentrations of TSH no significant stimulation of $^3$H-TdR incorporation occurred in suspension cultures throughout the 6-day culture period, the day 4–5 incorporation being 1,005 $\pm$ 114 cpm and 1,132 $\pm$ 35 cpm for TSH at 0.01 mU ml$^{-1}$ and at 10 mU ml$^{-1}$ respectively (Figure 1b). Similarly, no stimulation of the monolayer autoradiographic nuclear labelling index was seen with these concentrations of TSH – data not shown. (Increasing IGF-1 concentration above 10 ng ml$^{-1}$ did not lead to any further increase (or decrease) in the response to TSH).

*Adenoma follicles* Three out of 5 adenomas gave the pattern of response illustrated (for one case) in Figure 2a, b. In contrast to the normal epithelium, a single highly significant peak of $^3$H-TdR incorporation at day 4–5 of the suspension culture period was observed when TSH at 0.1 mU ml$^{-}$1 was present *alone* in the culture medium (without IGF-1), the $^3$H-TdR incorporation rising to 2,900 $\pm$ 200 cpm from a basal (no growth factor) incorporation of 450 $\pm$ 10 cpm ($P<0.001$). The corresponding monolayer autoradiographic nuclear labelling index rose from 2% to 12.6% (Figure 3b). However, TSH at lower (0.01 mU ml$^{-1}$) or higher (10 mU ml$^{-1}$) concentrations gave a much reduced response, the day 4–5 $^3$H-TdR incorporation being 580 $\pm$ 27 cpm and 900 $\pm$ 40 cpm respectively (Figure 2a). The inclusion of IGF-1 (10 ng ml$^{-1}$) did not significantly affect the response to TSH at 0.1 mU ml$^{-1}$, the day 4–5 $^3$H-TdR incorporation in suspension culture being 3,700 $\pm$ 900 cpm in the presence of IGF-1, compared with 2,900 $\pm$ 200 cpm in its absence (Figure 2a, b). The monolayer nuclear labelling indices were 10.7% and 12.6% respectively (Figure 3b).

The second and third adenomas showed a similar pattern of response, with TSH alone at 0.1 mU ml$^{-1}$ inducing single, highly significant peaks of $^3$H-TdR incorporation.

In a fourth adenoma, whilst TSH alone (at 0.1 mU ml$^{-1}$) was again stimulatory, the further addition of IGF-1 (at 10 ng ml$^{-1}$) resulted in a paradoxical inhibition of $^3$H-TdR incorporation, the day 4–5 value being reduced from 6,998 $\pm$ 600 cpm with TSH alone, to 1,115 $\pm$ 70 cpm with TSH plus IGF-1 ($P<0.001$).

Finally, a fifth adenoma showed an apparent 'spontaneous' $^3$H-TdR incorporation, occurring in the absence of any added growth factors. A peak occurred on day 2–3 of the culture period, reaching 3,380 $\pm$ 800 cpm. Addition of TSH at 0.1 mU ml$^{-1}$ with or without IGF-1 at 10 ng ml$^{-1}$ produced no significant stimulation or inhibition of this

Figure 1 Effect of TSH, (a) alone, or (b) in combination with IGF-1 on $^{3}$H-TdR incorporation in normal human thyroid follicular epithelium cultured in suspension as described in **Materials and methods**. (a) RPMI alone (○); TSH 0.01 mU ml$^{-1}$ (●); TSH 0.1 mU ml$^{-1}$ (▲); TSH 10 mU ml$^{-1}$ (■). (b) IGF-1 10 ng ml$^{-1}$ (○); IGF-1 10 ng ml$^{-1}$ + TSH 0.01 mU ml$^{-1}$ (●); IGF-1 10 ng ml$^{-1}$ + TSH 0.1 mU ml$^{-1}$ (▲); IGF-1 10 ng ml$^{-1}$ + TSH 10 mU ml$^{-1}$ (■).

**Figure 2** Effect of TSH, (a) alone, or (b) in combination with IGF-1 on $^{3}$H-TdR incorporation in adenomatous human thyroid follicular epithelium cultured in suspension. (a) RPMI alone (○); TSH 0.01 mU ml$^{-1}$ (●); TSH 0.1 mU ml$^{-1}$ (▲); TSH 10 mU ml$^{-1}$ (■). (b) IGF-1 10 ng ml$^{-1}$ (○); IGF-1 10 ng ml$^{-1}$ + TSH 0.1 mU ml$^{-1}$ (▲); IGF-1 10 ng ml$^{-1}$ + TSH 10 mU ml$^{-1}$ ■. [Data not available for 0.01 mU ml$^{-1}$ TSH.]

response, the day 2–3 incorporation being 2,750 ± 300 cpm and 2,400 ± 187 cpm respectively.

### Discussion

We have previously demonstrated that a proliferative response to TSH could be obtained in cultures of *normal* human thyroid follicular cells, but required the presence of a combination of two permissive factors, namely a supra-physiological level of insulin coupled with a low concentration of serum. This response was demonstrated in suspension cultures both by measurement of $^{3}$H-TdR incorporation by scintillation counting and by direct assessment of the nuclear labelling index by autoradiography, the latter being necessary to exclude artefacts arising from changes in the kinetics of thymidine uptake and synthesis (Williams *et al.*, 1987). However, in this earlier work, no response could be observed in parallel monolayer cultures of normal human follicular epithelium using the same combination of permissive factors.

Our present data now demonstrate that the permissive effect of insulin and serum in suspension cultures of normal human thyroid follicles can be substituted for by a physiological concentration of a single permissive factor, namely IGF-1, a combination of IGF-1 (at 10 ng ml$^{-1}$) and TSH (at 0.1 mU ml$^{-1}$) producing a highly significant peak of $^{3}$H-TdR incorporation occurring on day 4–5 of the culture period.

**Figure 3** Effect of TSH either alone, or in combination with IGF-1, on the nuclear labelling index of (a) normal and (b) adenomatous human thyroid follicular epithelium cultured as monolayers as described in **Materials and methods**. RPMI alone (○); TSH $0.1\,mU\,ml^{-1}$ (△); IGF-1 $10\,ng\,ml^{-1}$+TSH $0.1\,mU\,ml^{-1}$ (▲).

Furthermore, we now find that with this permissive factor, a proliferative response to TSH is also seen in *monolayer* cultures of normal thyroid follicular epithelium.

The TSH-induced proliferative response in both suspension and monolayer culture of normal follicular cells shows a bell-shaped concentration-effect curve with a clear response to TSH at $0.1\,mU\,ml^{-1}$ and loss of this response at $10\,mU\,ml^{-1}$. Such bell-shaped responses are a well-documented feature of *in vitro* studies of cell proliferation (Bodger *et al.*, 1979; Yen & Lewin, 1981) and have been previously reported in human thyroid follicular epithelium derived from normal glands, Graves' disease and multinodular goitre (Ollis *et al.*, 1986) although, interestingly, not in cultures of follicular epithelium derived from other mammalian species (Roger *et al.*, 1982; Valente *et al.*, 1983; Smith *et al.*, 1986).

For the comparison of normal and adenomatous follicular epithelium in this study, we have chosen to employ both suspension and monolayer modes of culture. Suspension cultures were used because there is both ultrastructural and functional evidence to support the view that they provide a more valid *in vivo* representation of the *in vitro* state of the follicular epithelium (Chambard *et al.*, 1983; Westermark *et al.*, 1986). Monolayers were used in order to facilitate comparisons between our study and those of other workers, nearly all of whom have employed this mode of culture.

Suspension culture autoradiography is an extremely time-consuming method of analysis. Since our previous work has always shown a close correlation between peaks of $^3$H-TdR incorporation, as measured by scintillation counting, and increases in the autoradiographic nuclear labelling index (Williams *et al.*, 1987; Smith *et al.*, 1986), the analysis of suspension cultures in this study was performed by $^3$H-TdR scintillation counting alone. However, all critical comparisons of growth responses were confirmed by use of autoradiographic nuclear labelling index in the monolayer cultures.

In cultures derived from follicular adenomas, it was found that 4 adenomas showed single peaks of $^3$H-TdR incorporation (occurring on day 4–5) in response to the addition of TSH *alone*. (Again, the TSH-induced proliferative response showed a bell-shaped concentration-effect curve with optimal stimulation occurring with TSH at $0.1\,mU\,ml^{-1}$ in both suspension and monolayer culture models). IGF-1 was without significant effect in 3 out of 4 of these adenomas, and was inhibitory in the fourth case. A fifth adenoma exhibited a spontaneous peak of $^3$H-TdR incorporation, the presence of either TSH and/or IGF-1 having no additional effect.

It is known that IGF-1 plays an important role in the mitogenic responses of diverse cell types (Van Wyk & Underwood, 1978). In rodent fibroblasts, for example, entry into S-phase of the cell-cycle is dependent upon the exposure of the cells to a tissue-specific 'competence' factor such as PDGF, together with or followed by a non- (or at least less-) tissue specific 'progression' factor such as IGF-1 (Stiles *et al.*, 1979; Leof *et al.*, 1982). Our *in vitro* model of normal thyroid follicular epithelial cell proliferation demonstrates an analogous requirement for both tissue-specific and non-tissue-specific growth factors, TSH and IGF-1 respectively, although no conclusions can be drawn as to their respective temporal roles in regulating entry into S-phase.

Selective loss of the requirement for IGF-1 in 4 out of 5 adenomas strongly suggests that escape from dependence on an exogenous source of IGF-1 confers a selective growth advantage on these tumour cells. The basis for this, however, is not immediately clear since IGF-1 is usually regarded as a permissive factor, produced by liver (D'Ercole *et al.*, 1984) and fibroblasts (Adams *et al.*, 1984), which is present at a fixed, non-limiting concentration in the extracellular fluid and it is assumed that thyroid growth is regulated only by the availability of the specific mitogen, TSH. Our results suggest, on the contrary, that the available concentration of IGF-1 may in fact be 'growth limiting' in the presence of normal TSH concentrations and that in adenomas elevation of the IGF-1 growth signal, achieved either by autocrine production of the growth factor itself or by activation of its intracellular messengers, permits a continued growth response to occur to a normal level of TSH thus leading to tumour formation. Evidence for autocrine IGF-1 production by neoplastic cells has, indeed, been documented in both cultures of neoplastic human breast epithelium (Huff *et al.*, 1986) and haemangiosarcoma (Pavelic *et al.*, 1986).

In conclusion, our results demonstrate for the first time a synergism between IGF-1 and TSH in the control of normal human thyroid follicular cell proliferation when cultured in suspension as closed follicles. Furthermore, we have documented major differences in the *in vitro* growth factor control of normal and adenomatous epithelium with 4 out of 5 adenomas exhibiting an apparent lack of requirement for exogenous IGF-1. We are currently exploring the possible mechanisms which have resulted in escape from IGF-1 requirement by these human tumour cells.

We are grateful to the Cancer Research Campaign for grant support.

### References

ADAMS, S.O., KAPADIA, M., MILLS, B & DAUGHADAY, W.H. (1984). Release of insulin-like growth factors and binding protein activity into serum-free medium of cultured human fibroblasts. *Endocrinology,* **115,** 520.

BODGER, M.P., McGIVEN, A.R. & FITZGERALD, P.H. (1979). Mitogenic proteins of pokeweed. 1, Purification, characterisation and mitogenic activity of two proteins from pokeweed (phytolacca octandra). *Immunology,* **37,** 785.

CHAMBARD, M., VERRIER, B., GABRION, J. & MAUCHAMP, J. (1983). Polarisation of thyroid cells in culture: Evidence for the basolateral localisation of the iodide 'pump' and of the thyroid-stimulating hormone receptor-adenyl cyclase complex. *J. Cell. Biol.,* **96,** 1172.

D'ERCOLE, A.J., STILES, A.D. & UNDERWOOD, L.E. (1984). Tissue concentrations of somatomedin C: Further evidence for multiple sites of synthesis and paracrine or autocrine mechanisms of action. *Proc. Natl Acad. Sci. USA.,* **81,** 935.

HUFF, K.K., KAUFMAN, D., GABBAY, K.H., SPENCER, E.M., LIPPMAN, M.E. & DICKSON, R.B. (1986). Secretion of an insulin-like growth factor-1 related protein by human breast cancer cells. *Cancer Res.,* **46,** 4613.

KLEIN, G. & KLEIN, E. (1985). Evolution of tumours and the impact of molecular oncology. *Nature,* **315,** 190.

LEOF, E.B., WHARTON, W., VAN WYK, J.J. & PLEDGER, W.J. (1982). Epidermal growth factor (EGF) and somatomedin C regulate $G_1$ progression in competent BALB/C3T3 cells. *Exp. Cell. Res.,* **141,** 107.

OLLIS, C.A., DAVIES, R., MUNRO, D.S. & TOMLINSON, S. (1986). Relationship between growth and function of human thyroid cells in culture. *J. Endocrinol.,* **108,** 393.

PAVELIC, K., VRBANEC, D., MARUSIC, S., LEVANAT, S. & CABRIJAN, T. (1986). Autocrine tumour growth regulation by somatomedin C: An *in vitro* model. *J. Endocrinol.,* **109,** 233.

ROGER, P.P., HOTIMSKY, A, MOREAU, C. & DUMONT, J.E. (1982). Stimulation by thyrotropin, cholera toxin and dibutyril cyclic AMP of the multiplication of differentiated thyroid cells *in vitro*. *Mol. Cell. Endocrinol.,* **26,** 165.

SMITH, P., WYNFORD-THOMAS, D., STRINGER, B.M.J. & WILLIAMS, E.D. (1986). Growth factor control of rat thyroid follicular cell proliferation. *Endocrinology,* **119,** 1439.

STILES, C.D., CAPONE, G.T., SCHER, C.D., ANTONIADES, H.N., VAN WYK, J.J. & PLEDGER, W.J. (1979). Dual control of cell growth by somatomedin and platelet derived growth factor. *Proc. Natl Acad. Sci. USA,* **76,** 1279.

VALENTE, W.A., VITTI, P., KOHN, L.D. & 6 others (1983). The relationship of growth and adenylate cyclase activity in cultured thyroid cells: Separate bioeffects of thyrotropin. *Endocrinology,* **112,** 71.

VAN WYK, J.J. & UNDERWOOD, L.E. (1978). The somatomedins and their actions. In *Biochemical Actions of Hormones,* Litwack, G. (ed) Vol. V, p. 101. Academic Press Inc., New York.

WESTERMARK, K., WESTERMARK, B., KARLSSON, F.A. & ERICSON, L.E. (1986). Location of epidermal growth factor receptors on porcine thyroid follicle cells and receptor regulation by thyrotropin. *Endocrinology,* **118,** 1040.

WILLIAMS, D.W., WYNFORD-THOMAS, D. & WILLIAMS, E.D. (1987). Control of human thyroid follicular cell proliferation in suspension and monolayer culture. *Mol. Cell. Endocrinol.,* **51,** 33.

WYNFORD-THOMAS, D., STRINGER, B.M.J. & WILLIAMS, E.D. (1982). Dissociation of growth and function in the rat thyroid during prolonged goitrogen administration. *Acta Endocrinol.,* **101,** 210.

YEN, A. & LEWIN, D. (1981). Uncoupling lymphocyte proliferation from differentiation: Dissimilar dose-response relations for pokeweed mitogen-induced proliferation and differentiation of normal human lymphocytes. *Cell. Immunol.,* **61,** 332.